# Innovative levers for sustainable integration of gender medicine into medical school curricula

**DOI:** 10.1186/s13293-016-0103-9

**Published:** 2016-10-14

**Authors:** Cara Tannenbaum, Geneviève Moineau

**Affiliations:** 1 The Canadian Institutes of Health Research, Institute of Gender and Health, Montreal, Canada; 2 The Association of Faculties of Medicine of Canada, Ottawa, Canada

**Keywords:** Accreditation levers, Gender medicine, Medical schools, Canada

## Abstract

Efforts to integrate gender medicine into medical school curricula have focused largely on the work of individual champions. Online sex and gender materials for undergraduate courses have also been developed and disseminated. Success has been sporadic, with varying uptake across schools within and between countries. International trends in medical school accreditation processes and the growing force of the millennial student voice offer untapped opportunities to promote more systematic integration of gender medicine on a national and international level. In this commentary, the president and CEO of the Association of Faculties of Medicine of Canada and the Scientific Director of the Institute of Gender and Health of the Canadian Institutes of Health Research jointly reflect on top-down and bottom-up levers for sustainable innovation in gender medicine for undergraduate medical training.

## Background

Suboptimal integration of gender medicine in medical school curricula has led to a call for more effective strategies to influence what medical students learn about managing sex differences and gender-related health issues [1, 2]. Several approaches have yielded pockets of success. Deployment of standardized content and gender medicine curricula online, support for faculty to develop gender medicine courses or programs locally, and advocacy to increase the assessment of gender medicine competencies in national medical licensing exams all offer hope for improvement [1–4]. However, modification of medical school curricula is a complex, multifaceted, and institutionalized process that extends beyond the scope of influence of local thought-leaders, or the introduction of innovative learning and evaluation methods for students. If the goal is significant and sustainable change in gender medicine education across countries to optimize high-quality and cost-effective care of patients, efforts to improve the status quo will need to adopt a systems-level perspective.

The Canadian Institutes of Health Research (CIHR) is committed to translating research knowledge into practice to help decision-makers, healthcare providers, and patients benefit from high-quality, evidence-based policies, programs, and services. The CIHR Institute of Gender and Health strives to identify opportunities to bridge the gap between sex and gender science and the health needs of men, women, boys, girls, and gender-diverse persons. To meet this objective, the CIHR Institute of Gender and Health invited the Association of the Faculties of Medicine of Canada to jointly reflect on pivotal system levers to enable large-scale, sustainable integration of evidence emerging from sex and gender research into undergraduate medical education. This piece discusses two promising drivers for improved training in gender medicine in university programs that educate and train students for certification as medical doctors and practitioners: trends in medical school accreditation and the student voice as an emerging catalyst for change.

### A systems level perspective: top-down accreditation pressure

Medical school accreditation provides a powerful incentive for revamping the content of the undergraduate curriculum. Accreditation is widely recognized as an important process by which designated authorities review and evaluate a medical school program using a specified set of standards to ensure that the minimum requirements are met to ensure and improve the quality and purpose of the program [5]. Each medical school goes through periodic review. National accreditation standards address quality in several categories, including curricular content. In Canada, for example, the Committee on the Accreditation of Canadian Medical Schools works with the Liaison Committee on Medical Education in the USA to ensure that Canadian medical faculty programs meet quality standards. A school can be placed on probation, which requires making changes to maintain accreditation. McGill medical school was put on probation in 2015, with 24 out of a total of 132 criteria found to be lacking [6]. Poor quality of instruction in women’s health was one factor, with students reporting they were receiving inadequate training in women’s health and family and domestic violence. Within 6 months, McGill responded with an action plan to address this and the other issues raised [7]. Being put on probation is rare; however, the threat of probation provides compelling motivation for medical schools to proactively ensure that their education program meets requirements.

An important strategy to drive better integration of gender medicine into medical school curricula involves approaching the committees that establish national medical school accreditation criteria. In Canada, presenting to the Committee at their Public Consultation held in conjunction with the Canadian Conference on Medical Education each spring would be a good place to start. The timing to implement this strategy is opportune. There is a global trend for medical schools to rethink their models and methods. The Association of Faculties of Medicine of Canada led the Health Canada funded Future of Medical Education in Canada MD project supported by all 17 medical schools [8]. In Europe, the move towards an international alignment of educational standards has potential to accelerate harmonization of gender medicine more rapidly than through individual local efforts [5]. Top-down pressure to conform would drive undergraduate deans and curriculum committees to impose development in gender medicine curriculum in order to meet evolving standards.

### Empowered students as catalysts of change: the bottom-up approach

The Millenial generation, born between 1982 and 2000, represents a new cadre of medical students who hold distinct perspectives about the world and are more engaged and vocal about social justice and gender equity than their generation X predecessors [9]. Both clinician educators and accreditation bodies recognize that students are best positioned to gauge the extent to which medical schools are meeting learners’ pedagogical needs and desired competencies [6, 10]. As students demand to learn more about gender issues, and speak up to the media about their concerns [11], medical schools will be encouraged to respond.

In Canada, at the end of their training, medical students complete the Association of Faculties of Medicine of Canada Graduation Questionnaire, which queries students’ perceptions on areas that were adequately or inadequately addressed during their time at the institution. This Graduation Questionnaire is used by accreditation bodies as an important source of information during each medical school site visit and assessment. In the 5 years preceding the 2015 accreditation at McGill, students reported inadequate instruction in women’s health (range 23.9–24.5 %) and family and domestic violence (range 51.5–59.1 %). When accreditors found no discussion on these particular topics at the new McGill curriculum executive level, the warning bell was sounded. As management requires measurement, inserting more detailed questions about instruction in sex differences and gender medicine competencies on the Graduation Questionnaire will improve the data platform that drives curriculum enhancement. An opportunity for students to present to the committee that formulates questions for the Graduation Questionnaire could be a channel for students to register their discontent about inadequacies in gender medicine. A combination of faculty champions and student advocates may be needed so that students are aware that they can actually approach the national committees with these requests. If medical students could be empowered to advocate in this manner, it would amplify the power of their collective voices beyond the local context.

## Conclusion

New top-down and bottom-up opportunities exist to integrate gender medicine into medical school curricula. Accreditation is a powerful lever which brings about sustainable change. Empowered students will have significant influence in shaping the gender medicine imperative in coming years. Using these approaches to strategically facilitate gender medicine integration is crucial to building a sex- and gender-specific evidence base into medical practice.
